# Pure Laparoscopic Left Hemihepatectomy for Hepatic Peribiliary Cysts with Biliary Intraepithelial Neoplasia

**DOI:** 10.1155/2016/7236427

**Published:** 2016-01-21

**Authors:** Akira Umemura, Takayuki Suto, Akira Sasaki, Hiroyuki Nitta, Seika Nakamura, Fumitaka Endo, Kazuho Harada, Kazuyuki Ishida

**Affiliations:** ^1^Department of Surgery, Morioka Municipal Hospital, 5-15-1 Motomiya, Morioka 020-0866, Japan; ^2^Department of Surgery, Iwate Medical University, 19-1 Uchimaru, Morioka 020-8505, Japan; ^3^Department of Anesthesiology, Morioka Municipal Hospital, 5-15-1 Motomiya, Morioka 020-8505, Japan; ^4^Department of Molecular Diagnostic Pathology, Iwate Medical University, 19-1 Uchimaru, Morioka 020-8505, Japan

## Abstract

*Introduction*. Hepatic peribiliary cysts (HPCs) usually originate due to the cystic dilatation of the intrahepatic extramural peribiliary glands. We describe our rare experience of pure laparoscopic left hemihepatectomy (PLLH) in a patient with HPCs accompanied by a component of biliary intraepithelial neoplasia (BilIN).* Case Presentation*. A 65-year-old man was referred for further investigation of mild hepatic dysfunction. Contrast-enhanced computed tomography showed dilatation of the left-sided intrahepatic bile duct, and biliary cytology showed class III cells. The patient was highly suspected of having left side-dominated cholangiocarcinoma and underwent PLLH. Microscopic findings revealed multiple cystic dilatations of the extramural peribiliary glands; hence, this lesion was diagnosed as HPCs. The resected intrahepatic bile duct showed that the normal ductal lumen comprised low columnar epithelia; however, front formation on the BilIN was observed in some parts of the intrahepatic bile duct, indicating that the BilIN coexisted with HPCs.* Conclusion.* We chose surgical therapy for this patient owing to the presence of some features of biliary malignancy. We employed noble PLLH as a minimally invasive procedure for this patient.

## 1. Introduction

Hepatic peribiliary cysts (HPCs) are a poorly recognized liver disease characterized by multiple tiny cysts along the portal radicle. HPCs usually originate due to the cystic dilatation of the intrahepatic extramural peribiliary glands around the intrahepatic large bile ducts [1]. Although HPCs sometimes cause stricture of the intrahepatic bile duct, they are also thought to have malignant potential for cholangiocarcinoma [2, 3].

Biliary intraepithelial neoplasia (BilIN), a microscopic biliary dysplasia with various cellular atypia, is one of the major intraductal neoplasms that can progress to invasive cholangiocarcinoma. Therefore, BilIN has to be accompanied by HPCs to progress to cholangiocarcinoma; however, there are no known reports of this process.

Laparoscopic liver resections have dramatically advanced in recent years but still remain a somewhat demanding modality for major liver resections. Pure laparoscopic major liver resection is therefore associated with concerns about intraoperative and postoperative morbidities [4, 5].

In this report, we describe our rare experience with pure laparoscopic left hemihepatectomy (PLLH) in a patient with HPCs accompanied by a component of BilIN.

## 2. Case Presentation

**Figure 1:**
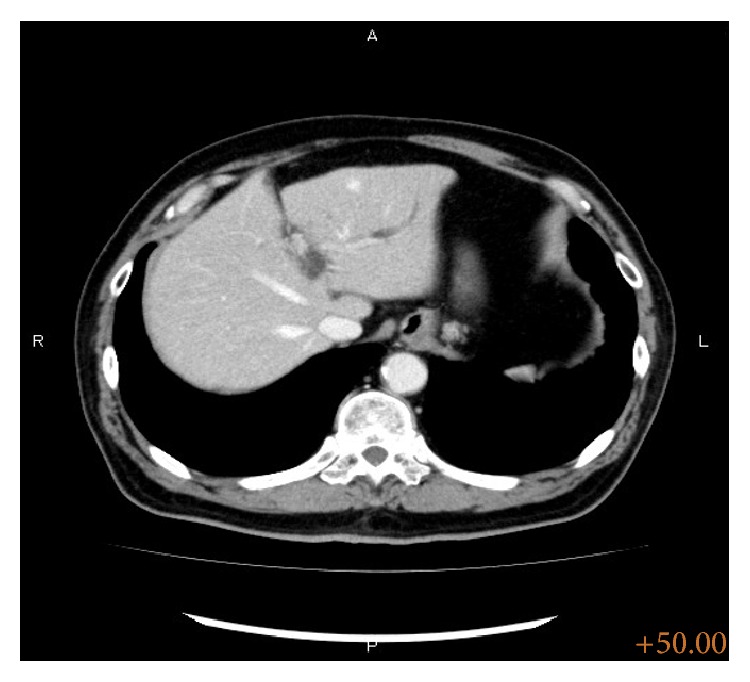
Enhanced CT examination revealed dilatation of the intrahepatic bile duct in the left hemiliver.

**Figure 2:**
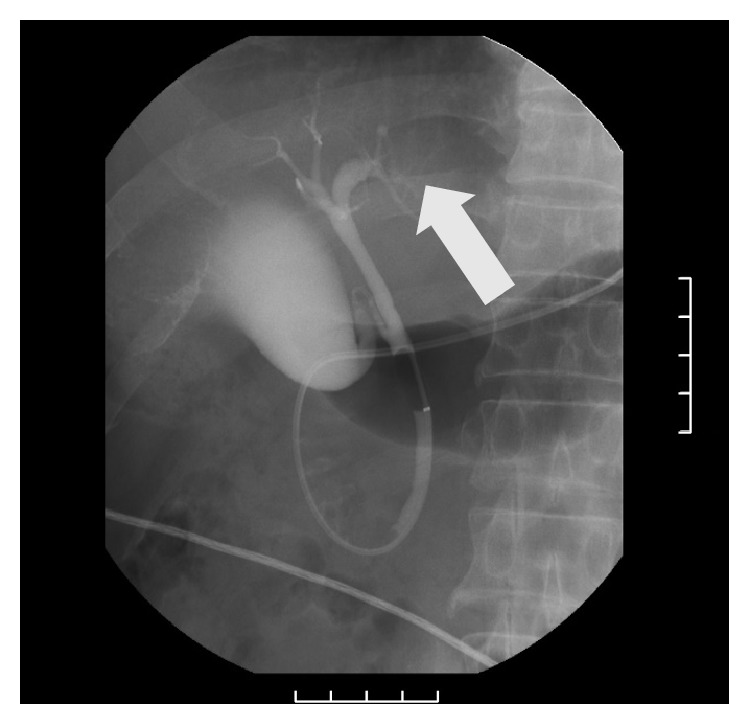
ERC revealed a filling defect within B3 (arrow).

A 65-year-old man was referred to our hospital for further investigation of mild hepatic dysfunction. Routine laboratory examination revealed liver dysfunction and increased tumor markers, with aspartate aminotransferase levels of 94 IU/L, alanine aminotransferase of 160 IU/L, carcinoembryonic antigen of 6.1 ng/mL, and carbohydrate antigen 19-9 of 129.1 U/mL. Contrast-enhanced computed tomography showed dilatation of the intrahepatic bile duct in the left hemiliver (Figure 1), and abdominal ultrasonography indicated the presence of a mass lesion in the left hepatic duct. In addition, endoscopic retrograde cholangiography revealed a filling defect within B3 (Figure 2), and biliary cytology showed class III cells. Based on these results, the patient was highly suspected of left side-dominated cholangiocarcinoma without hilar invasion; hence, we planned a laparoscopic surgical intervention with curative intent.

**Figure 3:**
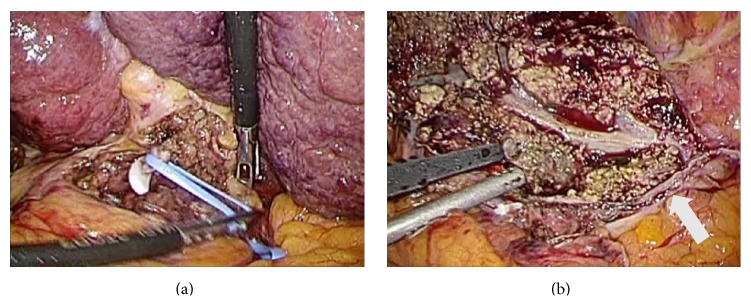
(a) The left branch of the portal vein was temporarily clamped so as to clarify a demarcation line. (b) The left hepatic vein was divided by an endoscopic linear stapler (arrow), and a cut surface along the middle hepatic vein was revealed.

The patient was placed in the right semilateral position under general anesthesia, and we used four port techniques in accordance with PLLH. During the hepatic parenchymal transection, the upper limit of the pneumoperitoneum pressure was set to 8–12 mmHg with monitoring of the central venous pressure. After the falciform and left triangular ligaments had been transected, cholecystectomy and regional lymphadenectomy were performed as usual. The left hepatic artery was then transected, and the left branch of the portal vein was dissected and temporarily clamped with a nonabsorbable clip (Hem-o-lok; Weck/Teleflex Medical, IL, USA) (Figure 3(a)). The demarcation line became clear, and intraoperative ultrasonography was performed to confirm the location of the middle hepatic vein in order to determine the surface cutting line of the liver. Hepatic parenchymal transection was performed using the clamp-crushing technique, employing a monopolar sealer (TissueLink Endo SH2.0; Salient Surgical Technologies, MN, USA) and a vessel-sealing system (ENSEAL; Ethicon Endo-Surgery, Tokyo, Japan). The left branch of the portal vein and the left hepatic duct were collectively divided with an endoscopic linear stapler (Echelon Flex 45 mm, open staple height 2.5 mm; Ethicon Endo-Surgery, Tokyo, Japan). Finally, the left hepatic vein was also divided with another endoscopic linear stapler (Figure 3(b)), and specimens were obtained from a 5 cm infraumbilical incision. An intraoperative frozen section at the stumps of the bile duct revealed a cancer-free surgical margin. The operating time was 214 min, and blood loss was 30 mL. A suction drain remained in place for 3 days. On postoperative day 13, the patient was discharged from the hospital.

**Figure 4:**
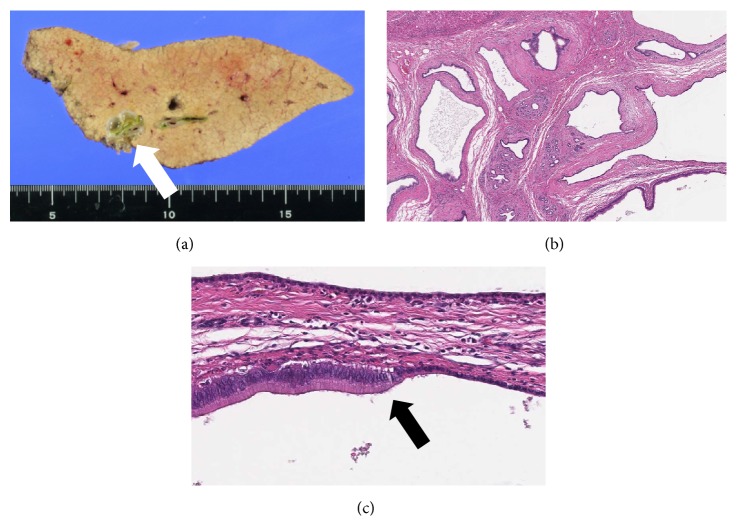
(a) Macroscopic findings of the resected specimens showed multiple cystic lesions surrounding the intrahepatic bile duct (arrow). (b) Multiple cystic dilatations of the extramural peribiliary glands with mucus production were observed (HE stain, ×40). (c) Front formation on the BilIN was observed in the intrahepatic bile duct (HE stain, ×400).

**Figure 5:**
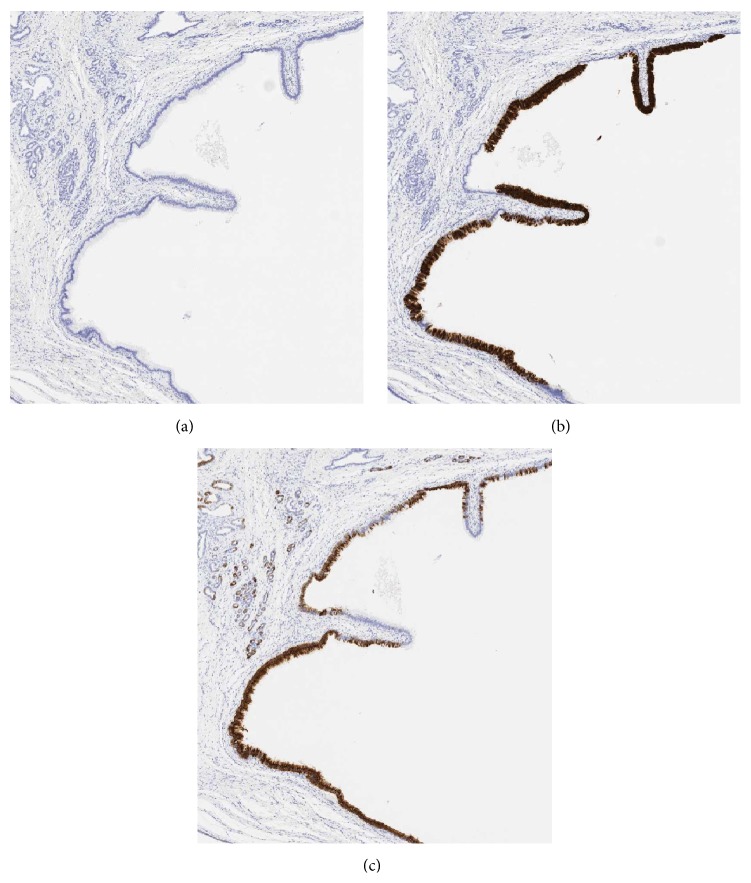
(a) Both the BilIN and background epithelia were negative for MUC2. (b) Only BilIN was positive for MUC5AC. (c) Not only BilIN but also the background epithelia were positive for MUC6.

Macroscopic findings of the resected specimens showed multiple cystic lesions surrounding the intrahepatic bile duct (Figure 4(a)). Microscopic findings using hematoxylin and eosin (HE) stain revealed multiple cystic dilatations of the extramural peribiliary glands with mucus production (Figure 4(b)); hence, this lesion was diagnosed as HPCs. The resected intrahepatic bile duct showed that the normal ductal lumen comprised low columnar epithelia; however, front formation on the BilIN was observed in some parts of the intrahepatic bile duct (Figure 4(c)). Immunohistochemical staining of the BilIN revealed that it was positive for mucin (MUC) 5AC and MUC6 and negative for MUC2 (Figures 5(a)–5(c)). The background epithelia were positive for only MUC6 (Figure 5(c)). From these results, BilIN was identified as coexisting with the HPCs. One year after the operation, the patient showed no signs of recurrence.

## 3. Discussion

HPCs originate from the cystic dilatation of the extramural peribiliary glands of the bile duct located in the connective tissue of the hepatic hilum and within the larger portal tract [1]. Owing to HPCs' characteristic localization, HPCs cause not only dilatation of the intrahepatic bile ducts, usually with multiple cystic tumors, but also obstructive jaundice and, on occasion, portal hypertension due to the exclusion of biliary and portal flow [6]. Therefore, it is often difficult to distinguish HPCs from cholangiocarcinoma, intraductal papillary neoplasm of the bile duct, or hepatic mucinous cystic neoplasm. With this clinical background, most HPCs have been surgically treated without accurate diagnosis.

To the best of our knowledge, HPCs are usually harmless. In addition, no cases of malignant degeneration from HPCs have been reported. Based on these reasons, we usually consider further surgical treatment to be unnecessary when HPCs are diagnosed accurately; however, there has been one report of HPCs accompanying bile duct carcinoma [3]. In addition, HPCs are usually detectable in preexisting hepatobiliary diseases, such as liver cirrhosis, alcoholic or nonalcoholic steatohepatitis, and polycystic kidney disease. HPCs are also known to develop in patients with inflammatory cystic dilatation of the biliary system after hepatic portoenterostomy, and chronic stimulation and inflammation caused by HPCs may influence the development of biliary malignancy [3, 6, 7]. Furthermore, BilIN may appear in the process of degeneration into invasive cholangiocarcinoma under these circumstances. If any findings suggest biliary malignancy with HPCs, surgical treatment via curative resection must be considered.

Depending on the surgical procedure, HPCs should be resected in a manner similar to that employed for other types of intrahepatic cholangiocarcinomas and extrahepatic bile duct carcinomas; therefore, major hepatectomy with or without extrahepatic bile duct resection or pancreaticoduodenectomy should be chosen. On the other hand, laparoscopic procedures for HPCs have not been described in detail, and laparoscopic resection for HPCs has not been reported until now. Major hepatectomies with biliary or vessel reconstruction are not presently indicated for laparoscopic procedures owing to the high rate of conversion and severe complications [4]. In addition, major laparoscopic liver resections are still globally limited to a few expert teams; however, we have performed pure laparoscopic liver resections and laparoscopy-assisted major liver resections on several occasions and have acquired sufficient experience in this field [5, 8]. Therefore, we performed PLLH safely in this patient by achieving adequate mobilization of the left lobe, taking enough measures to reduce bleeding upon liver resection, and selecting appropriate energy devices [5, 8].

## 4. Conclusion

In conclusion, HPCs may have the potential to complicate biliary malignancies, including BilIN; hence, a surgical procedure should be considered when some features of biliary malignancy are revealed with HPCs. We employed PLLH as a minimally invasive procedure for our patient. Although PLLH can have the clinical benefit of early postoperative recovery, PLLH requires adequate experience of laparoscopic liver resections to ensure safety during the surgical procedure.
